# SP1 Mediated PIK3CB Upregulation Promotes Gastric Carcinogenesis

**DOI:** 10.7150/jca.83812

**Published:** 2024-01-20

**Authors:** Kailing Fan, Qingqing Hu, Shijun Yu, Yong Gao, Yandong Li

**Affiliations:** 1 Department of Oncology, Shanghai East Hospital, School of Medicine, Tongji University, Shanghai 200120, China.; 2 Research Center for Translational Medicine, Shanghai East Hospital, School of Medicine, Tongji University, Shanghai 200120, China.

**Keywords:** SP1, PIK3CB, Gastric cancer, AKT, TGX-221

## Abstract

PIK3CB, one of catalytic subunits of PI3Ks kinase family, is implicated in several cellular processes such as cell growth, proliferation, mobility and neoplastic transformation. Its abnormal expression has been found in several human cancer types. However, the regulation pattern and function of PIK3CB in gastric cancer (GC) are still unclear. Here, we demonstrated that PIK3CB and SP1 (special protein 1) were both upregulated in GC samples compared to adjacent non-cancerous stomach tissues at mRNA and protein levels. The expression of the two genes also displayed a significant positive correlation in GC samples. Dual-luciferase assays and chromatin immunoprecipitation (ChIP) assays revealed that SP1 could bind to the -771~-605 region of the promoter of PIK3CB and enhance transcription. Furthermore, we discovered that SP1 induced AKT activation through PIK3CB and accelerated GC cell proliferation and migration in a PIK3CB/AKT signaling dependent manner. TGX-221, a PIK3CB-selective inhibitor, which can block this signaling transduction pathway, was found to inhibit the growth of GC cells and induce apoptosis *in vitro*, implying that it may act as a potential development agent for GC. These collective findings provide a new insight into PI3K/AKT signaling that SP1 may function as an upstream factor on PI3K, forming a new signaling axis to promote the progression of GC or other malignancies.

## Introduction

To date, Gastric cancer (GC) is still one of the most common and deadly malignancies. According to the Global Cancer Statistics 2020 1, GC ranks the fifth of newly diagnosed cases and the fourth death rate globally. Due to lack of early screening methods, early gastric cancer is difficult to detect and lots of people are diagnosed with advanced or metastatic stage of GC. On the other hand, owing to high recurrence and metastasis, the overall treatment effect of GC is poor with less than one year median survival time 2. Therefore, it is still necessary to develop new biomarkers and therapeutic targets for the treatment of this cancer.

Intracellular signal regulation network in cancer cells is complex. Among them, PI3K/AKT pathway is characterized to be constitutively activated in various cancers including GC 3-5. Deregulation of important factors in this pathway may affect various biological processes such as tumor cell growth, metabolism, and angiogenesis 6-8. It is universally acknowledged that oncogenic activation of PI3Ks or loss function of PTEN is the critical event during tumor initiation and progression 9,10. Class IA PI3Ks are the most significantly correlated with human malignancies. Three known catalytic subunits of Class IA PI3Ks, p110α, p110β and p110δ are encoded by three independent genes PIK3CA, PIK3CB and PIK3CD. PI3KCA is frequently mutated in human cancer, which results in constitutive activation of PI3K 11,12. PIK3CB and PI3KCD usually exhibit high expression and amplification in clinical cancer samples 13-16. Several groups also reported gain-of-function mutations in PIK3CB. PIK3CB has gained more attention in recent years for which more studies proved that PTEN-deficient cancers depend on PIK3CB, not PIK3CA 17,18. However, the regulatory mechanism and function of PIK3CB in GC are still to be unexplored.

SP1 (specificity protein 1) is a well-known member of SP transcription factor family, which contains three highly homologous C_2_H_2_-type zinc fingers in its C-terminal and prefers to bind the same GC consensus site 19,20. SP1 used to be viewed as a constitutive transcription factor and in charge of a wide range of so-called housekeeping gene expression. Tumor development is now known to be affected by SP1 in a dual manner, both activating expression of oncogenes as well as tumor suppressors 21. Earlier study has revealed abnormal SP1 protein level in GC, which is related to tumor stage and poor prognosis 22. The current studies have confirmed that SP1 could bind to the promoter region thus activate transcription of its downstream genes to promote tumor progression 23-25.

In this study, we determined the expression level of PIK3CB and SP1 by online database and verified their expression correlation in GC specimens. Mechanistically, we investigated the role of SP1 in transcription regulation of PIK3CB and activation of AKT kinase. In addition, we explored the effects of a PIK3CB specific inhibitor TGX-221 on GC cell proliferation and migration. We aimed to suggest a new SP1/PIK3CB/AKT signaling axis involved in tumorigenesis.

## Materials and methods

### Cell culture and reagents

Gastric cancer cell lines used in this study (AGS, SGC7901, NCI-N87, HGC-27, MGC823 and MGC803) were obtained from Shanghai Cell Bank of Chinese Academy of Sciences. Cells were cultured in DMEM medium supplemented with 10% FBS, penicillin (100 U/ml), and streptomycin (100 U/ml). All cells were cultured in a 37°C with 5% CO_2_. PIK3CB inhibitor TGX-221 was supplied by Med Chem Express (HY-10114) and the working concentration was at 10 μM.

### Human GC tissue specimens and immunohistochemical analysis

Forty paired GC tumor and their adjacent normal tissues used for qRT-PCR and related assays were collected from Shanghai East Hospital of Tongji University (Shanghai, China). Patients in this study who received any preoperative treatment were excluded and the diagnosis of GC was confirmed by pathological examination. Adjacent normal tissues were taken from over 5 cm distance from primary tumors. These tissues were preserved at -80°C before using. The human GC tissue microarray containing 48 gastric cancer tissues and adjacent normal tissues were purchased from Zhuoli Biotech (Shanghai, China). To detect the expression pattern of PIK3CB and SP1 in GC samples, standard immunohistochemical staining procedures were performed using a specific antibody against SP1 (Cell Signaling Technology, 9389S, 1:100) and PIK3CB (Cell Signaling Technology, 3011S, 1:100). The use of human clinical samples in this study was approved by the Medical Ethics Committees of Shanghai East Hospital, Tongji University.

### RNA extraction and quantitative real-time polymerase chain reaction (qRT-PCR)

The total RNAs were extracted from cultured GC cells or GC tissue specimens with Trizol reagent (Invitrogen, USA) according to the manufacturer's instructions, and then the concentration of RNA was quantified by a Fisher Scientific NanoDrop™ 2000 spectrophotometer (NanoDrop; Thermo Fisher Scientific, USA). RNA was reverse-transcribed into cDNA using the PrimeScript™ RT reagent kit (Takara Japan). cDNA was then used to perform Standard qRT-PCR analysis by ABI QuantStudioTM 6 Flex system (Thermo Fisher Scientific) using SYBR green reagent (TaKaRa, Japan) in a final volume of 10 μL. Relative mRNA expression of SP1 and PIK3CB were determined by 2^-ΔΔCt^ method using their threshold cycle (CT) values and β-actin was considered as an internal control. The following primers for gene amplification were used:

SP1-Forward: 5′-TGGCAGCAGTACCAATGGC-3′; SP1-Reverse: 5′-CCAGGTAGTCCTGTCAGAACTT-3′; PIK3CB-Forward: 5′-CCTCTCAGAACTCTATGTTGAA-3′; PIK3CB-Reverse: 5′-ATCCTGTCGTAAATCATCAC-3′; β-actin-Forward: 5'-CCTGGCACCCAGCACAATG-3'; β-actin-Reverse: 5'-GGGCCGGACTCGTCATACT-3'.

### Plasmid construction, RNA inference and lentivirus transfection

To overexpress SP1 and PIK3CB in GC cell lines, a full-length cDNA encoding SP1 (NM_138473.3) or PIK3CB (NM_006219.3) was firstly PCR-amplified and inserted into pcDNA 3.1 vectors. Empty pcDNA3.1 vector was used as a control. Two different siRNAs target different region of SP1 mRNA (siSP1-1 and siSP1-2), one siRNA target PIK3CB mRNA (siPIK3CB) and negative control siRNA (siNC) were purchased from GenePharma (Shanghai, China). The sequences are as follows:

siSP1-1: 5'-UGGUGGUGAUGGAAUACAUTT-3'; siSP1-2: 5'-CUGGUCAAAUACAGAUCAUTT-3'; siPIK3CB: 5'-GGGAAAGAGAACUGAGUAUTT-3'; siNC: 5'-UUCUCCGAACGUGUCACGUTT-3'.

Cell transfection was carried out by using Lipofectamine 3000 Transfection Reagent (Invitrogen, CA, USA). To obtain stable SP1-knockdown (shSP1) and PIK3CB-knockdown (shPIK3CB) cell lines, lentiviral particles containing shRNA against SP1 (shSP1) or PIK3CB (shPI3KCB) with sequence used in siSP1-1 and siPIK3CB were purchased from Gene Pharma biotech (Shanghai, China). GC cell lines were transfected with lentivirus constructed above with Polybrene (5 μg/ml) at a confluence of 80% host cells. After 48 h, stable infected cells were selected by puromycin for at least one week and gene knockdown efficiency were determined by western blot.

### Cell proliferation assay

The viability of GC cells was assessed using the Cell Counting Kit-8 (CCK-8) assay kit (Dojindo, Kumamoto, Japan). Briefly, GC cells were firstly transfected with specific siRNA or plasmids. After 48h of incubation, cells (3000 cells/well) were resuspended and seeded into 96-well plates. The optical density (OD) values of each well were measured at a wavelength of 450 nm by SpectraMax M5 for 5 days. Then cell growth curves were presented by GraphPad Prism 8.2.1. The half maximal inhibitory concentration (IC_50_) of GC cells was measured by the same kit of cell viability assay. Cells (5000 cells/ well) were seeded into 96-well plates. After cell spreading, cells were treated with different dose of TGX-221. After 48 h incubation, the OD values at 450 nm were measured. The colony capability of GC cells was conducted by colony formation assay, cells (1000 cells/well) were seeded and cultured with different dose of TGX-221 for at least 14 days. After fixed with paraformaldehyde, cells were then stained with crystal violet until cell were all stained. The images colonies were photographed and counted using Image J software.

### Cell migration assay

The migration ability of GC cell lines was assessed by transwell chamber assay. GC cells were transfected with siRNA against SP1 or SP1-overexprssion plasmid or PIK3CB-overexprssion plasmid, and after 24 hours cells were resuspended into single cell suspension and counted. About 3×10^4^ cells were seeded onto the upper chambers, which is incubated without FBS. The 24-well plates were filled with DMEM contains 5% FBS as chemoattractant. After incubation for 1-2 days, non-invading cells were wiped off with a cotton swab from the upper chamber. The rest cells were fixed by paraformaldehyde and stained with Crystal Violet. Cells that migrated through the chambers were photographed by microscope. These images were taken in at least five random fields and the average number of five fields was calculated.

### Protein extraction and western blot analysis

The total protein of GC cells was extracted by using RIPA lysis buffer with a protease inhibitor or phosphorylated protease inhibitor (Sigma Aldrich, Darmstadt, Germany). Western blotting was performed according to standard protocol with these antibodies and dilutions: anti-SP1 (Cell Signaling Technology, 9389S, 1:500); anti-PIK3CB (Cell Signaling Technology, 3011S, 1:800); anti-p-AKT (Cell Signaling Technology, 9271S, 1:500); anti-AKT (Cell Signaling Technology, 9272S, 1:500); anti-β-actin (Proteintech, 20536-1-AP, 1:3000). The proteins expression level was finally visualized using the Odyssey Infrared Imaging System.

### Dual luciferase reporter assay

We cloned a 908-bp fragment of the PIK3CB promoter and inserted it into the pGL3-basic luciferase reporter vector to create PIK3CB promoter luciferase reporter plasmid (WT) and a mutant construct at SP1 putative binding site was also generated by mutagenesis approach in this reporter plasmid (MUT). To conduct this assay, GC cells were seeded and transfected with the indicated promoter reporter plasmid (500 ng) and pRL-SV40 plasmid (20ng). 24 hours after transfection, cells were lysed by Dual-Glo® Luciferase Assay Kit (Promega, WI, USA) according to the manufacturer's instructions and the luciferase activities were measured by GloMax® 96 Microplate Luminometer (Promega, WI, USA).

### Chromatin immunoprecipitation (ChIP) assay

The exponentially growing AGS cells (1x10^7^) were prepared for the ChIP assay using ChIP assay kit (Millipore, CA, USA) according to the manufacturer's instructions. The finally collected DNA fragments were analyzed by PCR to amplify the binding region of the PIK3CB-promoter. P4+P5:167 bp; P2+P3: 314 bp; P1+P6: 307 bp.

The primers used in the ChIP assay were listed as follows:

P1: 5'-CTGGCAACGGTAGAGGAATCG-3' (sense); P2: 5'-CTATCCTCACAAAGGGAGTG-3' (sense); P3: 5'-CTCACTGCCGATTCCTCTAC-3' (antisense); P4: 5'-CTCTGCAGACAAATGTTAGG-3' (sense); P5: 5'-TCACTCCCTTTGTGAGGATA-3' (antisense); P6: 5'-CATCCCTGCCTCTCTCGATCA-3' (antisense).

### Flow cytometry

To observe cell apoptosis rate, AGS and HGC-27 cells were firstly treated with TGX-221(10 μM) for 24 h or 48 h. Cells were then collected and stained using an apoptosis detection kit (Dojindo, Kumamoto, Japan) according to the manufacturer's instructions. Fluorescence was measured using a flow cytometer.

### Statistical analysis

Quantitative data are presented as mean ± standard deviation and statistical analysis were performed using GraphPad Prism 8.2.1 software. Student's t-test was used to determine the significance of the quantitative data and P <0.05 were considered statistically significant. Linear regression was used to analyze the relationship between SP1 expression and PIK3CB expression.

## Results

### PI3KCB and SP1 are co-overexpressed in GC tissues

**Figure 1:**
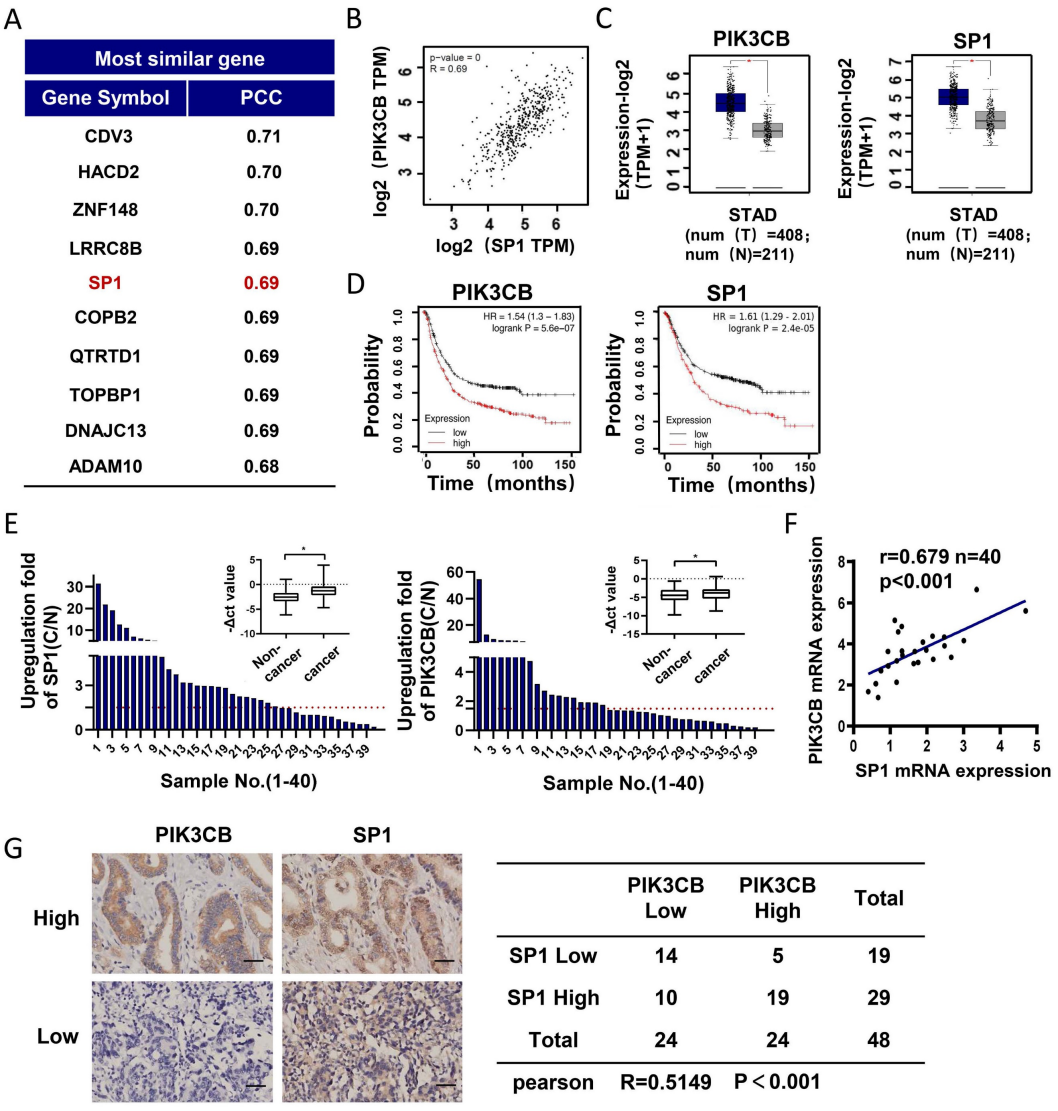
** PIK3CB and SP1 were overexpressed in GC samples.** (A) The top 10 genes co-expressed with PIK3CB in TCGA-STAD datasets were shown. PCC: Pearson correlation coefficient. (B) The expression relevance between PIK3CB and SP1 was predicted by GEPIA online tools. (C) PIK3CB and SP1 were upregulated in GC tissue samples form TCGA database using GEPIA. (D) Kaplan-Meier curves for patients with different expression levels of PIK3CB or SP1 via Kaplan-Meier plotter database. (E) qRT-PCR analysis of PIK3CB and SP1 expression in 40 paired GC tissue and adjacent normal tissues. C/N score ≥1.5 was viewed as upregulated. (F) The assessment of correlation between PIK3CB and SP1 expression in 40 paired GC tissues using linear regression. (G) PIK3CB and SP1 expression level were evaluated by immunohistochemistry staining on a GC tissue microarray. Representative staining images and expression correlation of PI3KCB and SP1 in tumor tissue were shown. Scale bar: 50 μm. *P < 0.01.

To explore the regulatory mechanism of PIK3CB in GC development, we employed GEPIA online analysis website (http://gepia2.cancer-pku.cn) to explore the genes co-expressed with PIK3CB in TCGA-STAD (The Cancer Genome Atlas Stomach Adenocarcinoma) data collection. As shown in Figure 1A, SP1 was found and ranked in the top 5. There was a strong positive relationship between PIK3CB and SP1 expression (Figure 1B, r=0.69, P < 0.01). In contrast to normal adjacent tissues, the mRNA levels of PIK3CB and SP1 showed notably higher in GC tissues (Figure 1C), suggesting the potential oncogenic activity of PIK3CB and SP1 in GC. Analysis of Kaplan-Meier plots for overall survival (http://kmplot.com/analysis) demonstrated that patients with higher PIK3CB or SP1 expression level had shorter overall survival (Figure 1D). To validate the results from online database analysis, we examined the expression of PIK3CB and SP1 in a cohort of 40 paired GC and non-tumor tissues by qRT-PCR. The result confirmed the upregulation of PIK3CB and SP1 in human GC tissue (Figure 1E). Meanwhile, the expression of PIK3CB was positively related to SP1 in these GC tissue samples (Figure 1F). Furthermore, we also examined the protein levels of PIK3CB and SP1 by immunohistochemical staining in two GC tissue microarrays containing 48 GC tissues. As expected, the two proteins both exhibited strong staining in most of samples (PIK3CB, 24/48; SP1, 29/48). A positive link between PIK3CB and SP1 was also observed at protein level (Figure 1G). These data suggested that PIK3CB and SP1 are co-overexpressed in GC and may be involved in GC development.

### SP1 transcriptionally regulates PIK3CB expression in GC cells

**Figure 2:**
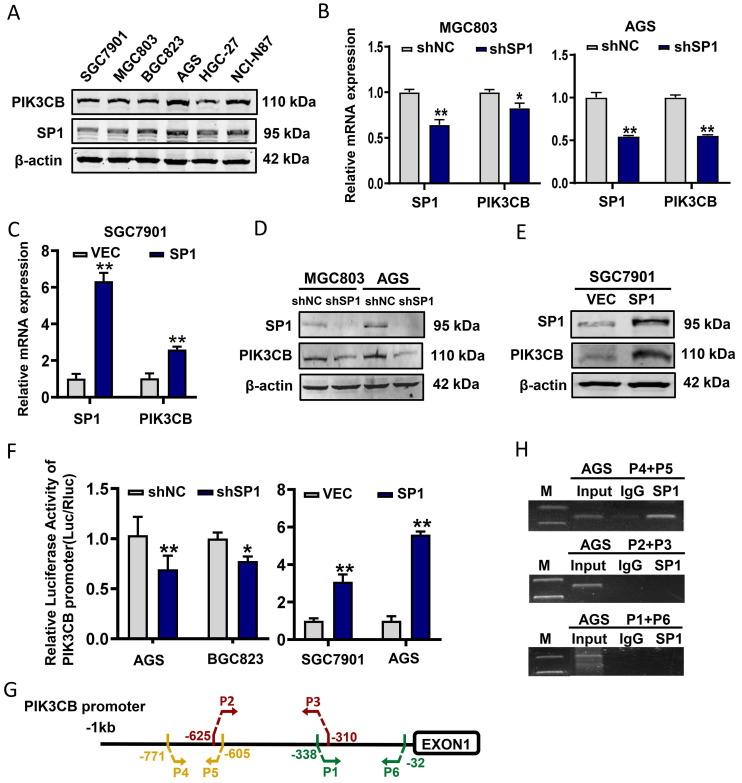
** PIK3CB expression was transcriptionally activated by SP1 in GC cells.** (A) The expression pattern of PIK3CB and SP1 were examined in a series of GC cell lines by western blot. (B) The mRNA level of PIK3CB was detected by qRT-PCR in SP1 stable knockdown cells. (C) SP1 overexpression by transient transfection upregulated the expression of PIK3CB by qRT-PCR analysis. (D) The protein level of PIK3CB was detected by western blot in SP1 stable knockdown cell lines. (E) SP1 overexpression by transient transfection upregulated the expression of PIK3CB by western blot. (F) The PIK3CB promoter reporter activities were examined in SP1 silenced or overexpressed GC cells as indicated. (G) Schematic illustration of the promoter region of PIK3CB. Primers designed to detect SP1 binding regions were indicated. (H) Chromatin immunoprecipitation assays were performed in AGS cells with specific SP1 antibody and control IgG. DNA fragment were amplified by PCR using P4+P5, P2+P3, P1+P6 primer. M: Marker. *P < 0.05, **P < 0.01.

Next, endogenous PIK3CB and SP1 expression levels in GC cell lines were examined by western blot analysis. The data slightly indicated a similar expression pattern of these two proteins in GC cell lines (Figure 2A). Subsequently, stable knockdown of SP1 in MGC803 and AGS cells were conducted by lentivirus-mediated shRNA infection. qRT-PCR and western blot results demonstrated that PIK3CB was markedly decreased with SP1 knockdown (Figure 2B, D). Concordantly, overexpression of SP1 by transiently transfecting SP1 plasmid to SGC7901 cells significantly increased PIK3CB expression (Figure 2C, E). To confirm whether SP1 directly regulates PIK3CB expression, we generated a luciferase construct containing a 908-bp PIK3CB promoter. Dual-luciferase reporter assays demonstrated that the relative luciferase activity of PIK3CB promoter was decreased with SP1 knockdown while elevated with SP1 overexpression (Figure 2F). ChIP assays were carried out in AGS cells and PCR results indicated that SP1could bind to the region (from -771 to -605) of the promoter in PIK3CB (Figure 2G, H). Based on the online JASPAR prediction tool (https://jaspar.elixir.no/) for SP1 binding site, we found a putative binding sequence within -771 to -605 region of PIK3CB promoter (Supplementary Figure 1A, B). The site mutation strongly suppressed SP1-induced promoter activity of PIK3CB (Supplementary Figure 1C). Taken together, these results demonstrated that SP1 could transcriptionally activate PIK3CB in GC cells.

### SP1 enhances AKT activity dependent on PIK3CB in GC cells

**Figure 3:**
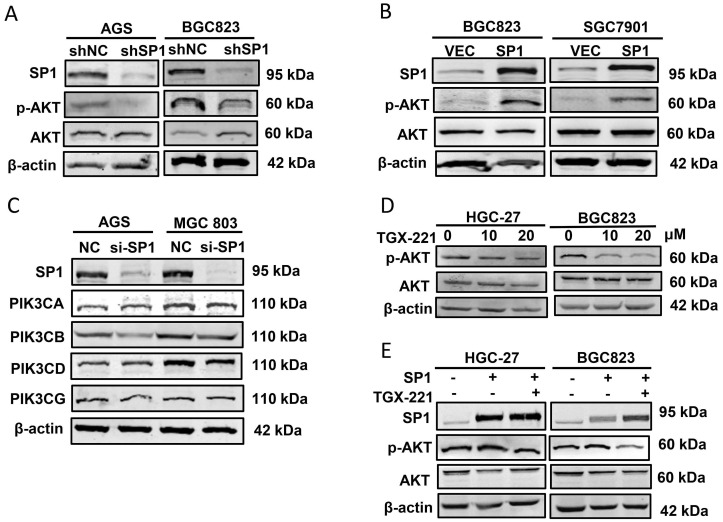
** PIK3CB mediates AKT activation by SP1.** (A) Western blot analysis confirming the effect of SP1 knockdown on p-AKT level in GC cells. (B) p-AKT levels were determined by western blot upon SP1 overexpression. (C) The effects of SP1 knockdown on four kinds of PI3K isoforms. (D) The protein level of p-AKT in GC cells was measured after treatment with different concentrations of TGX-221. (E) Western blot analysis of p-AKT level in HGC-27 and BGC823 cells with or without SP1 overexpression, or treatmenbt with or without TGX-221 (20μM). p-AKT, Ser473 phosphorylated AKT.

Since SP1 affects PIK3CB expression, we further addressed whether SP1 can regulates AKT phosphorylation that represents the activation of PI3K/AKT pathway. Western blot results showed that p-AKT (Ser473 site) was decreased in SP1 silenced AGS and BGC823 cells (Figure 3A). In parallel, p-AKT was upregulated when SP1 was overexpressed in BGC823 and SGC7901 cells (Figure 3B). To exclude the other isoforms of PI3Ks involved in this regulation, we assessed the protein expression of PIK3CA, PIK3CB, PIK3CD and PIK3CG (encoding another isoform of Class IB PI3K catalytic subunit, p110γ) by western blot analysis. The data revealed that only PIK3CB expression was both obviously decreased in both GC cells tested (Figure 3C). TGX-221, a PIK3CB-sepecific inhibitor, could block PIK3CB kinase activity and AKT activation. Western blot results verified that TGX-221 treatment significantly decreased p-AKT levels (Figure 3D). More importantly, AKT activation induced by SP1 overexpression could be attenuated upon TGX-221 treatment (Figure 3E). These results implied that SP1 regulates p-AKT level via PIK3CB.

### TGX-221 suppresses growth and induces apoptosis of GC cells* in vitro*

**Figure 4:**
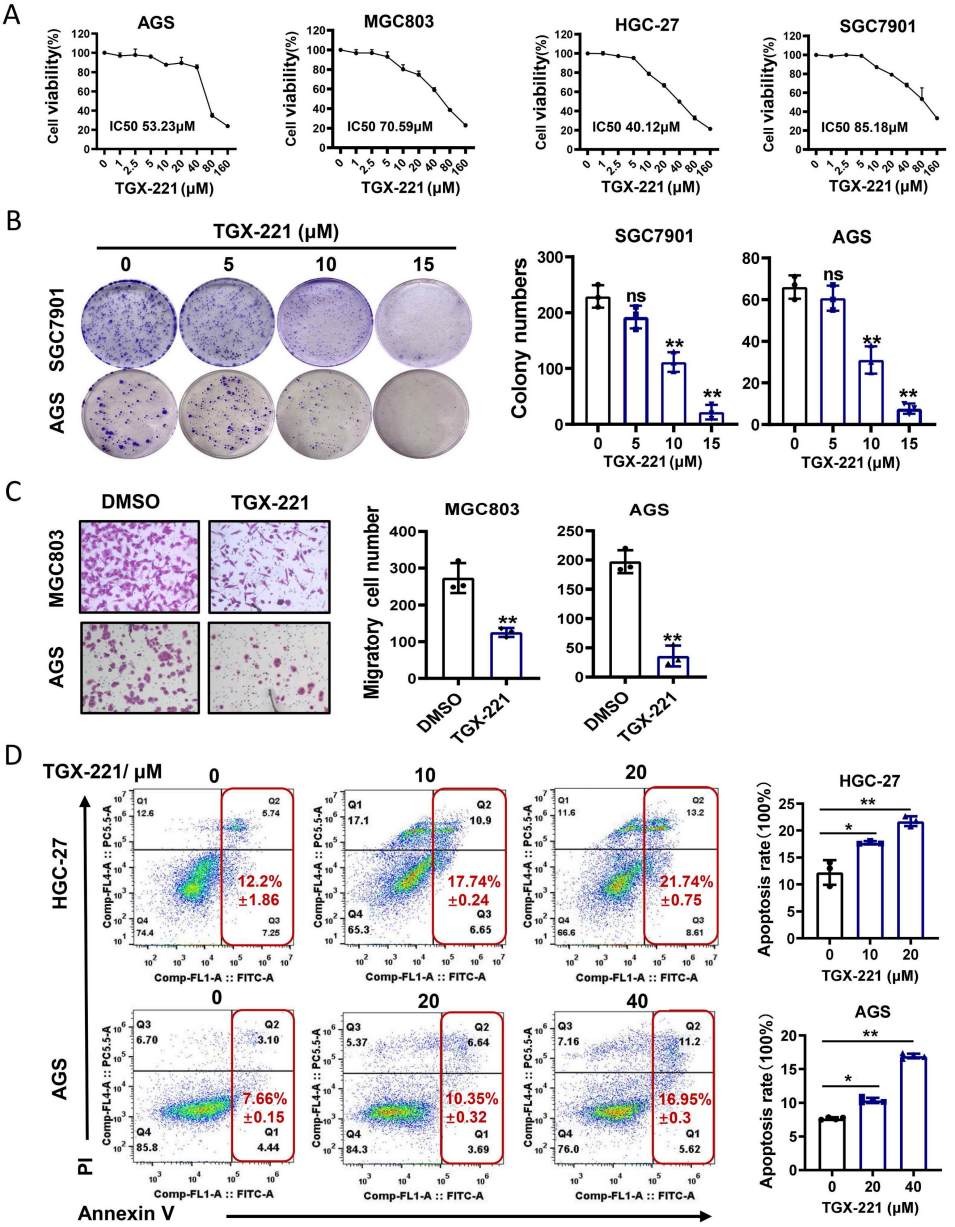
** TGX-221 can serve as a potential agent for GC therapy.** (A) Dose-response curves of GC cell lines treated with different concentrations of TGX-221 and measured at 48 h. IC50, half-maximal inhibitory concentration. (B) The effects of proliferation ability of TGX-221 on GC cells determined by colony formation. (C) The effects of migration ability of TGX-221 on GC cells determined by transwell chamber assay. (D) Apoptosis cells were analyzed by flow cytometry after 24 h (HGC-27) or 48h (AGS) after treatment with TGX-221 at indicated concentrations. *P < 0.05, **P < 0.01, ns, no statistical significance.

TGX-221 was developed upon detailed structure and function analysis of LY294002, a well-documented inhibitor of pan-PI3K/AKT pathway. However, whether TGX-221 exerts suppressive effects in GC has not been studied. To this end, cell survival analysis was carried out under the treatment of TGX-221 in GC cells. The results indicated that a significant dose-dependent reduction in cell viability with the dose of TGX-221 increasing (Figure 4A). Interestingly, HGC-27, a loss of PTEN function cell line, showed the most sensitive to the treatment of TGX-221. The colony formation and migration abilities of GC cells were also significantly inhibited by TGX-211 (Figure 4B, C). To further evaluate the impact of TGX-221 on cell apoptosis, we performed flow cytometry assay. As a result, TGX-221 treatment showed more significant apoptotic rates in AGS and HGC-27 cells (Figure 4D). These findings hinted that TGX-221 may serve as a potential anticancer agent for GC.

### PIK3CB is required for SP1-mdeidated GC cell growth and migration

**Figure 5:**
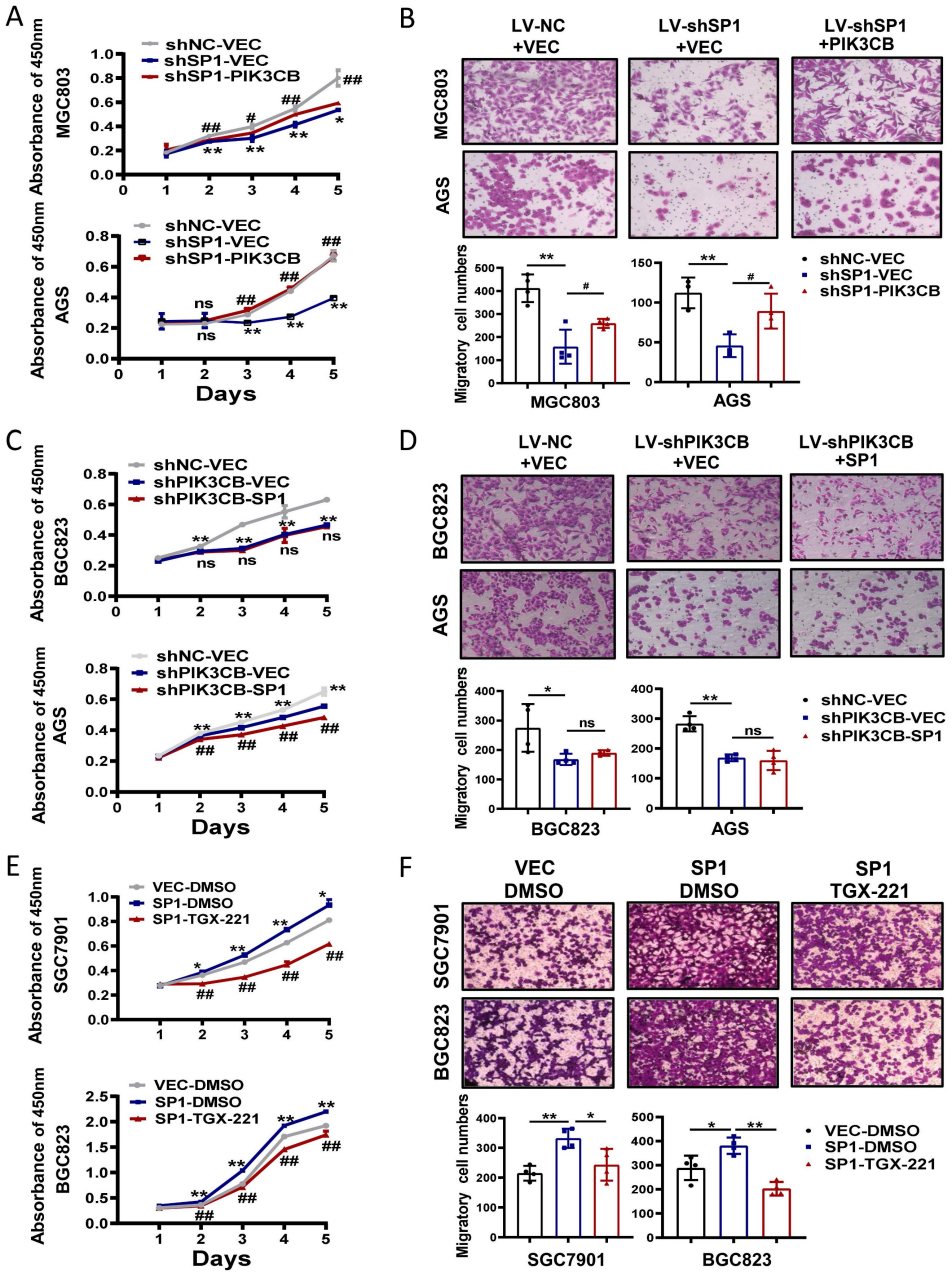
** The functional relationship between SP1 and PIK3CB in GC cell growth and migration.** (A, B) Cell viability assays (A) and transwell assays (B) were performed in SP1 stable knockdown cells with or without PIK3CB overexpression as indicated. (C, D) The effects of SP1 overexpression on cell proliferation (C) and migration (D) in PIK3CB silenced cells or control cells were examined by CCK-8 and transwell assays. (E, F) TGX-221 treatment had repressive effects on SP1-mediated cell proliferation (E) and migration (F) in SGC7901 and BGC823 cells. TGX-221: 10 μM. *P < 0.05, **P < 0.01, ns, no statistical significance.

To investigate the possible function link between SP1 and PIK3CB, we firstly examined the effects of PIK3CB overexpression in SP1 stable knockdown cells through CCK-8 and transwell chamber assays. PIK3CB could restore the phenotype defects caused by SP1 knockdown as indicated in Figure 5A, B. On the other hand, PIK3CB silencing decreased GC cell growth and migration, and the phenotypes were not reversed when SP1 plasmid was transfected into PIK3CB stable knockdown cells (Figure 5C, D). The similar results were observed when PIK3CB silencing replaced with TGX-221 (Figure 5E, F). Collectively, these results revealed that SP1 could induce cell proliferation and migration dependent on PIK3CB at least in part.

## Discussion

PIK3CB, an isoform of PI3Ks kinase family, is reported to have a pivotal regulatory role in cell growth and tumorigenesis, which is differ from PIK3CA 26. PIK3CB overexpression has been described in pancreatic cancer and colorectal cancer 27-29. Our current work for the first time provides evidence that PIK3CB is also overexpressed in GC and patients with higher expression of PIK3CB is correlated with poor prognosis. We used bioinformatics database TCGA to find co-expressed genes with PIK3CB. The transcription factor SP1 aroused our interest based on its important roles reported in several human cancers 19,20. Our findings illustrate the concomitant expression of PIK3CB and SP1 in GC samples and cells. Following ChIP and luciferase reporter assays we show that SP1 binds to the -771~-605 region of the promoter of PIK3CB. These results suggest that PIK3CB may serve as a downstream effector of SP1 and supply one of possible explanations for the overexpression of PIK3CB in GC.

There is previously no transcription factor being reported to regulate PIK3CB expression in cancers or other diseases. We also confirm that SP1 is positively corelated with AKT activity. Overexpression of SP1 contributed to an increase in p-AKT level while knockdown of SP1 decreased p-AKT level. By conducting a range of functional experiments, we observed that SP1 induces the proliferation and migration of GC cells through regulating PIK3CB. Given that there is no research about the role of PIK3CB in GC, our findings provide novel regulation pathway of SP1/PIK3CB/AKT axis in promoting GC development. Interestingly, previous studies described that both the expression of SP1 and its active form p-SP1 (Ser453) can be regulated by AKT kinase 30,31. Thus, we made an attempt to address whether activated AKT kinase could affect the expression of SP1 and p-SP1 (S453) by western blotting. However, inconsistent with those results, neither the expression of SP1 nor p-SP1 has been upregulated by the transfection of activated-AKT plasmid (data not shown). This inconsistence may be required for further investigations in future.

Based on previous studies, PI3K/AKT pathway has been reported to play a crucial role in the progression of GC, as such, it may possibly provide potential target for targeted therapy. TGX-221 is the first identified PIK3CB isoform-selective inhibitors, which was developed upon the function and structure of LY294002^32^. The operating principle of TGX-221 is to function as a competitive inhibitor, targeting the ATP-binding site of the kinase domain. It is reported that TGX-221 has an IC_50_ value of 5nM against purified kinase and have an important role in antithrombotic activity by inhibiting platelet aggregation 32. Several studies have indicated that TGX-221 inhibit cell growth and promote apoptosis 33,34. As well, our results also provide evidence that TGX-221 could inhibit cell proliferation and migration of GC cells and induce cell apoptosis, suggesting it may be a potential effective drug for GC. The limitation of our research is lacking the *in vivo* data in mouse models or a combination therapy through dual targeting PIK3CB and other kinase targets.

In summary, our findings demonstrated that PIK3CB and SP1 were co-overexpressed in gastric cancer. We highlighted a new regulatory mechanism of GC development, which SP1 transcriptionally regulates PI3KCB and PIK3CB activates AKT signaling, thereby contributing to GC cell growth and migration. Finally, we provided anticancer agent TGX-221 may serve as a promising therapeutic strategy for GC.

## Supplementary Material

Supplementary figure.

## Abbreviations

SP1: Specificity protein 1
GC: Gastric cancer
CCK8: Cell Counting Kit
siRNA: small interfering RNA
qRT-PCR: Quantitative real-time PCR
TMA: Tissue Microarray
ChIP: Chromatin immunoprecipitation
GEPIA: Gene Expression Profiling Interactive Analysis
TCGA: The Cancer Genome Atlas
IHC: immunohistochemical staining
