# Supplementary material for: SP1 Mediated PIK3CB Upregulation Promotes Gastric Carcinogenesis
Source: J Cancer. 2024 Jan 20;15(5):1355–65. doi: 10.7150/jca.83812 (PMC10861831; doi:10.7150/jca.83812)
Supplement: Supplementary file 1 — Supplementary figure. [file jcav15p1355s1.pdf]

Supplementary Figure 1. SP1 promotes PIK3CB promoter activity via putative binding site 5’-TAGGTGTTGT-3’.

(A) The actual logo used for the searches for SP1 binding via online JASPAR prediction tool (https://jaspar.elixir.no/). (B) The putative binding site of SP1 in the promoter of PIK3CB. Red nucleotides represent the sequence mutations in the luciferase reporter construct. (C) The PIK3CB promoter reporter activities were examined in AGS cells with WT (wild-type promoter of PIK3CB) or MUT (mutated promoter of PIK3CB) reporter plasmid. SP1 and control vector was transiently transfected into AGS cells. $*P < 0.05$, $**P < 0.01$.

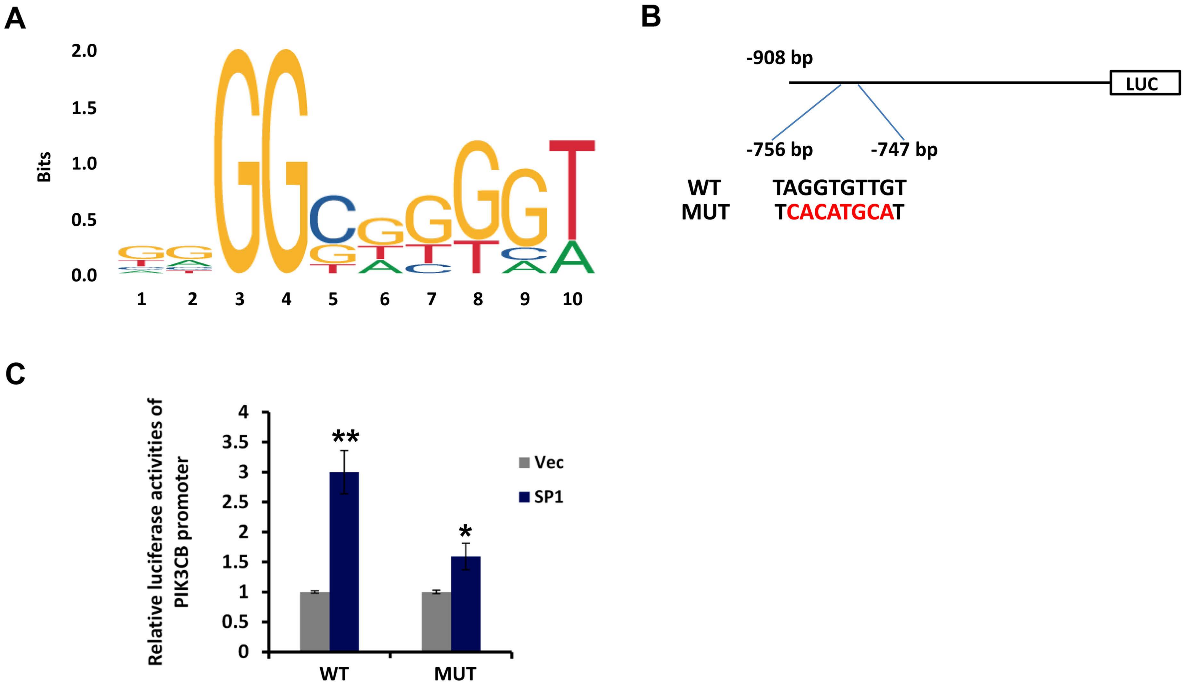

A
2.0
1.5
1.0
0.5
0.0
Bits
1
2
3
4
5
6
7
8
9
10
B
-908 bp
LUC
-756 bp
-747 bp
WT TAGGTGTTGT
MUT TCACATGCAT
C
Relative luciferase activities of PIK3CB promoter
4
3.5
3
2.5
2
1.5
1
0.5
0
**
*
Vec
SP1
WT
MUT
